# Sample size determination for training set optimization in genomic prediction

**DOI:** 10.1007/s00122-023-04254-9

**Published:** 2023-03-13

**Authors:** Po-Ya Wu, Jen-Hsiang Ou, Chen-Tuo Liao

**Affiliations:** 1 Department of Agronomy, National Taiwan University, Taipei, Taiwan; 2 Present Address: Institute for Quantitative Genetics and Genomics of Plants, Heinrich Heine University, Düsseldorf, Germany; 3 Present Address: Department of Medical Biochemistry and Microbiology, Uppsala University, Uppsala, Sweden

## Abstract

**Key message:**

A practical approach is developed to determine a cost-effective optimal training set for selective phenotyping in a genomic prediction study. An R function is provided to facilitate the application of the approach.

**Abstract:**

Genomic prediction (GP) is a statistical method used to select quantitative traits in animal or plant breeding. For this purpose, a statistical prediction model is first built that uses phenotypic and genotypic data in a training set. The trained model is then used to predict genomic estimated breeding values (GEBVs) for individuals within a breeding population. Setting the sample size of the training set usually takes into account time and space constraints that are inevitable in an agricultural experiment. However, the determination of the sample size remains an unresolved issue for a GP study. By applying the logistic growth curve to identify prediction accuracy for the GEBVs and the training set size, a practical approach was developed to determine a cost-effective optimal training set for a given genome dataset with known genotypic data. Three real genome datasets were used to illustrate the proposed approach. An R function is provided to facilitate widespread application of this approach to sample size determination, which can help breeders to identify a set of genotypes with an economical sample size for selective phenotyping.

**Supplementary Information:**

The online version contains supplementary material available at 10.1007/s00122-023-04254-9.

## Introduction

Genomic prediction (GP) has become increasingly popular for the selection of quantitative traits in animal or plant breeding programs since it was first proposed by Meuwissen et al. (2001). The main idea of GP is to capture quantitative trait loci using high-density molecular markers across an entire genome. Typically, a statistical prediction model is built by fitting trait values with the marker-associated scores for individuals in a training set. The resulting statistical model is then used to predict genomic estimated breeding values (GEBVs) for individuals of a breeding population. The GEBV of each individual is the fitted value produced by plugging its marker-associated scores into the statistical model. In practice, breeders can select superior individuals from the breeding population using their GEBVs (Heffner et al. 2010).

The construction of the statistical model plays a key role in breeding programs that use GP, and its prediction accuracy for GEBVs is highly dependent upon the data quality of the training set. The selection of an optimized training set can be a critical factor for accurate GEBV prediction (Zhong et al. 2009; Lorenz and Smith 2015; Zhang et al. 2019). Current genotyping costs have fallen dramatically, but phenotyping costs have remained relatively constant (Akdemir and Isidro-Sánchez 2019). Optimizing the training set for selective phenotyping can be an economical and efficient way to increase the chance of success in a genomic selection (Heslot and Feoktistov 2020). The training set size is limited by breeding resource budget constraints. Hence, the sample size of the optimal training set should be carefully chosen to balance trade-offs between prediction accuracy and phenotyping costs in a GP study. However, the determination of the sample size in training set optimization is still an unresolved issue (Isidro y Sanchez and Akdemir 2021). Practically speaking, the solution may require both the technical skills of statisticians and the scientific knowledge of breeders. There are three main statistical approaches to sample size determination: (i) determine the sample size to achieve a desired power of a hypothesis testing; (ii) determine the sample size to achieve a confidence interval of a specified width; (iii) determine the sample size to optimize a utility function that relates the estimation efficiency to sample size (Lenth 2001). To create a sample size determination of interest, we proceed by constructing a utility function that draws a connection between GEBV prediction accuracy and the size of the training set.

Methods of training set optimization can be classified into two categories. First is the untargeted method, which does not use genomic information from a target test set to determine the training set. Second is the targeted method, in which the training set is determined to maximize prediction accuracy for a target test set (Akdemir et al. 2015; Akdemir and Isidro-Sánchez 2019). Using the genomic best linear unbiased prediction (GBLUP) model, Rincent et al. (2012) compared several optimization criteria for the targeted method and then promoted a generalized coefficient of determination (CD) (Laloë 1993; Laloë et al. 1996) to determine an optimal training set. Isidro et al. (2015) and Rincent et al. (2017) extended CD-based optimization for highly structured populations. Most recently, Rio et al. (2022) proposed new versions of CD to forecast the GP reliability of genotypes. Using the whole genome regression (WGR) model, Akdemir et al. (2015) assessed prediction error variance (PEV) to optimize the training set for the targeted method. Both CD- and PEV-based methods can be easily modified to produce untargeted methods by replacing the target test set with the remaining set (individuals that are not selected into the training set) or the entire candidate set in the calculation of the criteria. Akdemir and Isidro-Sánchez (2019) compared the untargeted and targeted methods and found that the latter had a generally superior prediction accuracy to the former, mainly because the targeted method takes advantage of information on the genomic relationship between the training set and the test set. Some optimization approaches were proposed by Akdemir and Isidro-Sánchez (2019) based on the classical criteria in the context of optimal designs such as A-optimality and D-optimality.

Recently, Ou and Liao (2019) proposed a new criterion, called the *r*-score, to determine an optimal training set based on the WGR model. The *r*-score criterion was derived directly from Pearson’s correlation between GEBVs and phenotypic values for a test set. In the article, the authors showed that the prediction accuracy of the *r*-score-based method is usually competitive with those of the CD- and PEV-based methods because it takes into account both PEV and prediction bias. The curves presented in Ou and Liao (2019) and Wu et al. (2019) that describe the relationship between the *r*-score and the size of training sets randomly selected from the candidate set appeared to be an S-shaped growth curve. In other words, the curve begins at some fixed point and monotonically increases in *r*-score rate until it reaches an inflection point, and then, the rate decreases and asymptotically approaches some final value (Ratkowsky 1983). This observation motivates us to investigate the sample size for a GP study using an S-shaped growth curve as a utility function. The objective of this study is to develop a systematic procedure for determining the size of the training set. First, an S-shaped growth curve, called the logistic growth curve, was employed to fit the *r*-score versus training set size using genotypic data alone. Then, an operating curve for the sample size determination was obtained from the fitted *r*-score, relative to that of the entire candidate set. The operating curve allows a user to weigh the prediction ability of GEBVs and the sample size for selective phenotyping and then obtain a cost-effective optimal training set.

## Materials and methods

### Genome datasets

Three genome datasets were analyzed in this study.

### 44 K rice dataset

This dataset presented by Zhao et al. (2011) contains 413 accessions with 36,901 single nucleotide polymorphism (SNP) markers and 36 traits. The accessions were divided into five subpopulations and one admixed group. Only 375 accessions were found, with no missing phenotypic values among the following traits: brown rice seed area, brown rice volume, flag leaf length (FLL), flag leaf width, plant height (PH), seed length, and seed volume. SNPs with a calling rate < 0.9 and individuals with missing rate > 0.1 were removed from the dataset, leaving 31,401 SNPs for 367 accessions for further analyses. The SNP at each locus was coded as − 1, 0, or 1 for the homozygote of the minor allele, the heterozygote, and the homozygote of the major allele, respectively. After SNP coding, any missing locus at an individual was imputed by the average over all of the available values of the SNP.

### Tropical rice dataset

This dataset, presented by Spindel et al. (2015), contains 73,147 SNP markers and 363 elite breeding lines belonging to indica or an indica-admixed group. Phenotypic observations were carried out eight times in 2009–2012, once in the dry and once in the wet season each year, on grain yield, flowering time (FT), and PH, although PH data were not available for the wet season of 2009. Phenotypic values for 35 out of the 363 individuals were missing; therefore, the adjusted means of only 328 individuals were used in this example. The SNP coding was the same as that in the 44 K rice dataset.

### Soybean dataset

This dataset, presented by Stewart-Brown et al. (2019), contains 2647 SNP markers and 483 recombinant inbred lines with the best linear unbiased predictor (BLUP) values of oil content (OC), protein content (PRC), and yield (YLD). The BLUP values for each genotype were calculated to account for variation resulting from environmental factors and maturity. Individuals were classified into four subpopulations and one admixed group, where the admixed group was composed of individuals in Sets 9–11 and Sets 12–14 (see Table 1 in Stewart-Brown et al. 2019). Only a total of 401 individuals had BLUP values for all three of the traits. SNPs with missing rates > 0.1 and minor allele frequencies < 0.05 were filtered out, leaving 2376 SNPs for 401 individuals retained for further analyses. SNP coding was the same as in the above 44 K rice dataset.

**Table 1:** Parameters fixed in the study scenarios and in building the operating curves to determine the training set size for datasets

Dataset	Scenario^a^	Parameters^b^	Operating curve^c^
44 K rice	Fixed	$$n_{c} = 250, n_{0} = 50,75,100$$	$$n_{\min } = 25, n_{\max } = 225,$$$$\delta = 25,$$ *m* = 10
	Non-fixed	$$n_{c} = 317, n_{0} = 50$$	$$n_{\min } = 25, n_{\max } = 300,$$$$\delta = 25,$$ *m* = 10
Tropical rice	Fixed	$$n_{c} = 200, n_{0} = 50,75,100$$	$$n_{\min } = 25, n_{\max } = 175,$$$$\delta = 25,$$ *m* = 10
	Non-fixed	$$n_{c} = 278, n_{0} = 50$$	$$n_{\min } = 25, n_{\max } = 275,$$$$\delta = 25,$$ *m* = 10
Soybean	Fixed	$$n_{c} = 275, n_{0} = 50,75,100$$	$$n_{\min } = 25, n_{\max } = 250,$$$$\delta = 25,$$ *m* = 10
	Non-fixed	$$n_{c} = 301, n_{0} = 100$$	$$n_{\min } = 25, n_{\max } = 275,$$$$\delta = 25,$$ *m* = 10

^a^Fixed: the fixed candidate set scenario; Non-fixed: the non-fixed candidate scenario

^b^$n_{c}$: the candidate set size; $n_{0}$: the test set size

^c^$n_{\min }$: starting size of the search for the optimal training set; $n_{\max }$: maximal size of the search for the optimal training set; $\delta$: increment of the size; *m*: number of optimal training sets at each fixed size

### Genomic prediction models

The following three statistical models are commonly used in GP studies.

- Whole genome regression model

The WGR model can be described as follows:

1 $${\varvec{y}} = \mu {\mathbf{1}}_{n} + \varvec{X\beta } + {\varvec{\varepsilon}}$$

where ${\varvec{y}}$ is the vector of phenotypic values of length *n*;$\mu$ is the constant term; ${\mathbf{1}}_{n}$ is the vector of order *n* with all elements equal to 1; ${\varvec{X}}$ is a marker-associated matrix of the order $n \times p$; ${\varvec{\beta}}$ is the vector of marker-associated effects of length *p*; and ${\varvec{\varepsilon}}$ is the vector of random errors. Here, *n* is the number of individuals, and *p* is the number of marker-associated components. The ridge regression estimation for ${\varvec{\beta}}$ is given as:

2 $$\hat{\varvec{\beta }} = {\varvec{X}}^{T} \left( {{\varvec{XX}}^{T} + \lambda {\varvec{I}}_{n} } \right)^{ - 1} \left( {{\varvec{y}} - \hat{\mu }{\mathbf{1}}_{n} } \right)$$

where ${\varvec{I}}_{n}$ is the identity matrix of order *n*; $\lambda$ is a shrinkage parameter; and $\hat{\mu }$ is an estimate for $\mu$ which is treated as a known value. The marker-associated matrix ${\varvec{X}}$ can be (i) the original marker score matrix; (ii) the standardized marker score matrix; (iii) the principal component (PC) score matrix derived from (i); or (iv) the PC score matrix derived from (ii). For cases (i) and (ii), *p* is the number of markers, and *p* is the number of PCs used in the model for (iii) and (iv). In this study, we used the (iv) as the marker-associated matrix throughout the analysis.

(b) rrBLUP model

Under the assumption that both ${\varvec{\beta}}$ and *ϵ* follow a normal distribution in the WGR model of Eq. (1), denoted by ${\varvec{\beta}}\sim N\left( {{\mathbf{0}},\user2{ }\sigma_{\beta }^{2} {\varvec{I}}_{p} } \right)$ and ${\varvec{\varepsilon}}\sim N\left( {{\mathbf{0}},\user2{ }\sigma_{\varepsilon }^{2} {\varvec{I}}_{n} } \right)$, the logarithm of the joint probability density function $f{(}{\varvec{y}},\varvec{ \beta }{|}\mu ,{\varvec{X}})$ is maximized when

3 $${\varvec{\beta}} = {\varvec{X}}^{T} \left( {{\varvec{XX}}^{T} + \lambda^{*} {\varvec{I}}_{n} } \right)^{ - 1} \left( {{\varvec{y}} - \mu {\mathbf{1}}_{n} } \right)$$

with $\lambda^{*} = \frac{{\sigma_{\varepsilon }^{2} }}{{\sigma_{\beta }^{2} }}$. The formula in Eq. (3) was called as rrBLUP for ${\varvec{\beta}}$ (Endelman 2011) if $\lambda^{*}$ and $\mu$ are replaced with appropriate estimates. This is because it is in the form of the ridge regression estimation of Eq. (2).

(c) GBLUP model

The GBLUP model can be described as follows:

4 $${\varvec{y}} = \mu {\mathbf{1}}_{n} + {\varvec{g}} + {\varvec{\varepsilon}}$$

where ${\varvec{g}}$ denotes the vector of genotypic values for the individuals, assumed by ${\varvec{g}}\sim N\left( {{\mathbf{0}},\varvec{ }\sigma_{g}^{2} {\varvec{K}}} \right)$. Here, ${\varvec{K}}$ is considered the genomic relationship matrix for measuring similarity among individuals through the marker-associated matrix. Several forms were employed for ${\varvec{K}}$ in the context of GP (Forni et al. 2011; Rincent et al. 2012; Tsai et al. 2021). GBLUP model is equivalent to rrBLUP model, if ${\varvec{g}} = \varvec{X\beta }$; ${\varvec{K}} = \frac{1}{p}{\varvec{XX}}^{T}$; and $\sigma_{g}^{2} = p\sigma_{\beta }^{2}$.

### The marker-associated matrix

Let ${\varvec{X}}$ be the original marker scores matrix, and ${\varvec{M}}$ be the standardized marker score matrix. That is, $m_{ij} = \frac{{x_{ij} - \overline{x}_{j} }}{{s_{j} }}$, where $m_{ij}$ and $x_{ij}$ are the $\left( {ij} \right){\text{th}}$ elements of ${\varvec{M}}$ and ${\varvec{X}}$, and $\overline{x}_{j}$ and $s_{j}$ are the sample mean and the sample standard deviation for column *j* in ${\varvec{X}}$, for $i = 1, 2, \ldots , n$, and $j = 1, 2, \ldots , p$. Under the assumption that $n < p$, the spectral decomposition was performed on ${\varvec{M}}^{T} {\varvec{M}}$, producing ${\varvec{M}}^{T} {\varvec{M}} = \mathop \sum \nolimits_{i = 1}^{n} u_{i} {\varvec{q}}_{i} {\varvec{q}}_{i}^{T}$, where $u_{i}$ is a nonzero eigenvalue of the order $u_{1} \ge u_{2} \ge \cdots \ge u_{n} > 0$, and ${\varvec{q}}_{i}$ is the eigenvector of length *p*. The PC score matrix was then obtained as:

5 $${\varvec{L}} = {\varvec{MQ}}$$

with ${\varvec{Q}} = \left[ {{\varvec{q}}_{1} \vdots {\varvec{q}}_{2} \vdots \cdots \vdots {\varvec{q}}_{n} } \right]$. In this study, we used as many PCs as individuals in the dataset.

### The *r*-score criterion

Let $S_{c}$, $S_{t}$, and $S_{0}$ denote the candidate set, the training set, and the test set, respectively. In addition, let $n_{c}$, $n_{t}$, and $n_{0}$ be the respective numbers of individuals in $S_{c}$, $S_{t}$, and $S_{0}$. Moreover, let ${\varvec{X}}_{c}$, ${\varvec{X}}_{t}$, and ${\varvec{X}}_{0}$ denote their respective PC score matrices of the orders $n_{c} \times p$, $n_{t} \times p$, and $n_{0} \times p$. Based on the WGR model of Eq. (1) and the ridge regression estimation in Eq. (2) without considering the constant term $\mu$, Ou and Liao (2019) developed the *r*-score criterion, given as follows:

6 $$r{\text{-score}} = \frac{{q_{12} }}{{\sqrt {q_{1} q_{2} } }},$$

where

$$\begin{aligned} & q_{12} = {\text{Tr}}\left[ {{\varvec{X}}_{0}^{T} \left( {{\varvec{I}}_{{n_{0} }} - \overline{\varvec{J}}_{{n_{0} }} } \right){\varvec{X}}_{0} {\varvec{AX}}_{t} } \right], \\ & q_{1} = \left( {n_{0} - 1} \right) + {\text{Tr}}\left[ {{\varvec{X}}_{0}^{T} \left( {{\varvec{I}}_{{n_{0} }} - \overline{\varvec{J}}_{{n_{0} }} } \right){\varvec{X}}_{0} } \right], \\ & q_{2} = {\text{Tr}}\left[ {{\varvec{A}}^{T} {\varvec{X}}_{0}^{T} \left( {{\varvec{I}}_{{n_{0} }} - \overline{\varvec{J}}_{{n_{0} }} } \right){\varvec{X}}_{0} {\varvec{A}}} \right] + Tr\left[ {{\varvec{X}}_{t}^{T} {\varvec{A}}^{T} {\varvec{X}}_{0}^{T} \left( {{\varvec{I}}_{{n_{0} }} - \overline{\varvec{J}}_{{n_{0} }} } \right){\varvec{X}}_{0} {\varvec{AX}}_{t} } \right]. \\ \end{aligned}$$

Here, ${\text{Tr}}\left[ \cdot \right]$ denotes the trace of a square matrix;${\varvec{A}} = {\varvec{X}}_{t}^{T} \left( {{\varvec{X}}_{t} {\varvec{X}}_{t}^{T} + \lambda {\varvec{I}}_{{n_{t} }} } \right)^{ - 1} ;$ and $\overline{\varvec{J}}_{{n_{0} }}$ is the square matrix with all elements equal to $\frac{1}{{n_{0} }}$. The shrinkage parameter $\lambda$ is fixed at 1 in the calculation of the *r*-score. The robustness of $\lambda$ for the sample size determination will be discussed in the final section. Note that the computational cost can be reduced using the PC score matrices (Akdemir et al. 2015; Ou and Liao 2019).

### The logistic growth curve

The logistic growth curve was used to model the change of *r*-score with the training set size, which can be described as:

7 $$y = \frac{\alpha }{{1 + \exp \left( {\beta - \gamma x} \right) }}$$

where $y$ denotes the *r*-score, $x$ stands for the training set size, $\alpha$ is an unknown parameter related to the asymptote, parameter $\beta$ relates to the intercept on y-axis, parameter $\gamma$ relates to the rate at which the *r*-score changes from its initial value (determined by the magnitude of $\beta$) to its final value (determined by the magnitude of $\alpha$), and exp denotes the natural exponential function whose basis is Euler’s number, a mathematic constant approximately equal to 2.71828. For a given number of pairs of the *r*-score and training set size, the R function nls () (R Core Team 2019) was used to perform nonlinear least squares estimation for the parameters in the logistic growth curve model of Eq. (7).

### The study scenarios

For a given dataset, a subset of $n_{c}$ individuals was first selected at random as the candidate set $S_{c}$. A fixed number of $n_{0}$ individuals was then randomly selected from the remaining individuals as the test set $S_{0}$. This setting was called the fixed candidate set scenario. Conversely, $S_{0}$ was first determined by random sampling from the original dataset, and then, the remaining individuals were treated as $S_{c}$. The candidate set $S_{c}$ varied with the test set $S_{0}$ in this case, so this characteristic was called the non-fixed candidate set scenario. Both targeted and untargeted methods were analyzed in the two scenarios.

For a specific $S_{0}$, the targeted method searched optimal training set $S_{t}$ from $S_{c}$ to achieve the maximum *r*-score between $S_{0}$ and $S_{t}$. However, no target test set was specified for the untargeted method, and the optimal training set $S_{t}$ was therefore identified such that the *r*-score between $S_{c}$ and $S_{t}$ was maximized. In other words, ${\varvec{X}}_{c}$ (the marker-associated matrix of $S_{c}$), not ${\varvec{X}}_{0}$ (the marker-associated matrix of $S_{0}$), is used to calculate the *r*-score for the untargeted method. Furthermore, the following sampling rule was employed to determine the number of genotypes for each cluster in a candidate set with a strong population structure. For the targeted method, the number of genotypes selected from each cluster of the candidate set is proportional to the size of the cluster in the target test set. For the untargeted method, corresponding genotypes were selected proportional to the size of the cluster in the candidate set.

### Determining training set size

For each of the fixed and non-fixed candidate set scenarios, the following procedure was proposed to construct the desired operating curves to determine the sample size for both the targeted and untargeted methods.

*Step 1* For a given candidate set $S_{c}$ with ${\varvec{X}}_{c}$ and a specific test set $S_{0}$ with ${\varvec{X}}_{0}$ (instead of $S_{c}$ with ${\varvec{X}}_{c}$ for the untargeted method), we generated a number of optimal training set $S_{t}$ based on the *r*-score criterion at the training set size $n_{t}$ varying from $n_{min}$ to $n_{max}$ by an increment of $\delta .$ That is, we repeatedly generated $m$ optimal training sets and obtained their resulting *r*-scores, for $n_{t}$ = $n_{\min } , n_{\min } + \delta , n_{\min } + 2\delta , \ldots , n_{\max } .$ Note that there is only one training set available for $n_{t}$ = $n_{c}$.

*Step 2* For the resulting *r*-scores and $n_{t}$ generated from Step 1, we performed the R function nls () to obtain the parameter estimates in the logistic growth curve model of Eq. (7).

*Step 3* Let *r*-score ($n_{t}$) $= \frac{{\hat{\alpha }}}{{1 + \exp \left( {\hat{\beta } - \hat{\gamma }n_{t} } \right) }}$ denote the resulting logistic growth curve. Then, the fitted *r*-score ($n_{t}$) at $n_{t}$ relative to the fitted *r*-score ($n_{c}$) at $n_{c}$ is given by:

8 $${\text{RErs}}\left( {n_{t} } \right) = \frac{{1 + \exp \left( {\hat{\beta } - \hat{\gamma }n_{c} } \right) }}{{1 + \exp \left( {\hat{\beta } - \hat{\gamma }n_{t} } \right) }}.$$

The ${\text{RErs}}\left( {n_{t} } \right)$ curve of Eq. (8) is a function of $n_{t}$ conditioned by $n_{c}$, so it can be implemented to determine the size of the training set. Note that ${\text{RErs}}\left( {n_{t} } \right)$ ranges from 0 to 1, representing the relative *r*-score using an optimal training set of size $n_{t}$ to the whole candidate set $S_{c}$ of size $n_{c}$. Accordingly, a user can easily obtain a cost-effective training set $S_{t}^{*}$ with size $n_{t}^{*}$ at an acceptable ${\text{RErs}}\left( {n_{t}^{*} } \right)$, where ${\text{RErs}}\left( {n_{\min } } \right) \le {\text{RErs}}\left( {n_{t}^{*} } \right) \le 1$.

### Validation of the procedure

For a given $S_{t}^{*}$, the GBLUP model given below was used to predict GEBVs for individuals in the test set $S_{0}$

9 $${\varvec{y}}_{t} = \mu {\mathbf{1}}_{{n_{t}^{*} }} + {\varvec{g}}_{t} + {\varvec{\varepsilon}}_{t} ,$$

where ${\varvec{y}}_{t}$ denotes the vector of phenotypic values in $S_{t}^{*}$; ${\varvec{g}}_{t}$ is the vector of genotypic values for $S_{t}^{*}$; and ${\varvec{\varepsilon}}_{t}$ is the vector of random errors. It is assumed that ${\varvec{g}}_{t} \sim N\left( {{\mathbf{0}},\sigma_{g}^{2} {\varvec{K}}_{t} } \right),$ where ${\varvec{K}}_{t} = \frac{1}{n}\left( {{\varvec{L}}_{t} {\varvec{L}}_{t}^{T} } \right)$ with ${\varvec{L}}_{t}$ being the submatrix of ${\varvec{L}}$ in Eq. (5) corresponding to $S_{t}^{*}$. Accordingly, the BLUP for ${\varvec{g}}_{t}$ and the best linear unbiased estimate (BLUE) for $\mu$ can be obtained from Henderson’s mixed-model equations (Henderson 1975). Let $\hat{\varvec{g}}_{0}$ be the BLUP for $S_{0}$, and let ${\varvec{K}}_{0}$ be the genomic relationship matrix between $S_{0}$ and $S_{t}^{*}$. From Henderson (1977),

10 $$\hat{\varvec{g}}_{0} = {\varvec{K}}_{0} ({\varvec{K}}_{t} )^{ - 1} \hat{\varvec{g}}_{t} ,$$

where $\hat{\varvec{g}}_{t}$ is the BLUP for ${\varvec{g}}_{t}$. The GEBVs for $S_{0}$ were predicted to be of the form $\hat{\varvec{g}}_{0}$ plus $\hat{\mu }$ (the BLUE for $\mu$). The Bayesian reproducing kernel Hilbert space (RKHS) method in the R package BGLR (Perez and de los Campos 2014) was used to obtain the GEBVs.

Subsequently, Pearson’s correlation between the resulting GEBVs and the phenotypic values recorded in the original dataset, denoted by $r\left( {n_{t}^{*} } \right)$, was calculated as a measure for the prediction ability using $S_{t}^{*}$. The corresponding Pearson’s correlation using $S_{c}$ can be similarly obtained, which is denoted by $r\left( {n_{c} } \right)$. The relative prediction ability of $S_{t}^{*}$ to $S_{c}$ is given as:

11 $${\text{REpa}}\left( {n_{t}^{*} } \right) = \frac{{r\left( {n_{t}^{*} } \right)}}{{r\left( {n_{c} } \right)}}.$$

Here, ${\text{REpa}}\left( {n_{t}^{*} } \right)$ was treated as a point estimate for ${\text{RErs}}\left( {n_{t}^{*} } \right)$. A box plot of the ${\text{REpa}}\left( {n_{t}^{*} } \right)s$ obtained from a number of repetitions was used to validate the above procedure to determine the sample size.

The parameters of $n_{c}$ and $n_{0}$ fixed in the study scenarios, and those of $n_{\min }$, $n_{\max }$, $\delta$, and *m* fixed in building the operating curves to determine the training set size are summarized in Table 1. ${\text{RErs}}\left( {n_{t}^{*} } \right)$ in Eq. (8) was fixed at 0.95 and 0.99 to produce the training set size $n_{t}^{*}$, and then, the optimal training set $S_{t}^{*}$ corresponding to $n_{t}^{*}$ were generated. For each setting of the parameters, the procedure was repeated 30 times. Note that the sampling rule set for the highly structured population was taken into account in the training set optimization for the 44 K rice and soybean datasets.

## Results

The fitted logistic growth curves and operating curves are displayed in Figs. S1–S6 of the Supplementary Materials, which showed that almost all of the observed data points were located on or quite close to the fitted logistic growth curves in the panels of each figure. However, there were still some relatively large deviations in the case of the targeted method under the non-fixed candidate set scenario in the 44 K rice dataset.

The mean and standard deviation of the resulting 30 optimal training set sizes ($n_{t}^{*}$) for each trait in the datasets are separately displayed in Tables 2, 3 and 4. Note that the untargeted method in the fixed candidate set scenario resulted in a unique $n_{t}^{*}$ at a given ${\text{RErs}}\left( {n_{t}^{*} } \right)$ for all of the 30 repetitions, because the fixed candidate set was used as the test set for calculating the *r*-score. Hence, there is only one operating curve for this case. The test set was not used to determine the sample size of training set for the untargeted method. The untargeted method therefore gave only $n_{t}^{*}$ regardless of $n_{0}$.

**Table 2:** Means and standard deviations (in parentheses) of the resulting training set sizes over 30 repetitions at ${\text{RErs}}\left( {n_{t}^{*} } \right) = 0.95$ and 0.99, under the fixed candidate set scenario with three different test set sizes ($n_{0} =$ 50, 75 and 100) and the non-fixed candidate set scenario with test $n_{0} = 50$, for both the targeted and untargeted methods in the 44 K rice dataset

Scenario	Method	$n_{0}$	${\text{RErs}}\left( {n_{t}^{*} } \right) = 0.95$	${\text{RErs}}\left( {n_{t}^{*} } \right) = 0.99$
Fixed candidate set	Targeted	50	31.22 (3.15)	106.27 (7.23)
		75	37.00 (2.44)	108.03 (4.85)
		100	39.90 (1.47)	110.47 (2.98)
	Untargeted		138 (NA)	214 (NA)
Non-fixed candidate set	Targeted	50	42.03 (7.52)	147.77 (16.64)
	Untargeted	50	164.53 (1.61)	264.43 (1.41)

**Table 3:** Means and standard deviations (in parentheses) of training set sizes over the 30 repetitions at ${\text{RErs}}\left( {n_{t}^{*} } \right) = 0.95$ and 0.99 under the fixed candidate set scenario with three different test set sizes ($n_{0} =$ 50, 75, and 100) and the non-fixed candidate set scenario with test $n_{0} = 50$ for both the targeted and untargeted methods in the tropical rice dataset

Scenario	Method	$n_{0}$	${\text{RErs}}\left( {n_{t}^{*} } \right) = 0.95$	${\text{RErs}}\left( {n_{t}^{*} } \right) = 0.99$
Fixed candidate set	Targeted	50	109.60 (3.69)	165.07 (2.98)
		75	111.93 (2.98)	166.33 (2.17)
		100	112.87 (1.83)	166.97 (1.38)
	Untargeted		131 (NA)	177 (NA)
Non-fixed candidate set	Targeted	50	146.87 (8.71)	226.40 (6.93)
	Untargeted	50	172.17 (1.46)	239.80 (1.00)

**Table 4:** Means and standard deviations (in parentheses) of the resulting training set sizes over 30 repetitions at ${\text{RErs}}\left( {n_{t}^{*} } \right) = 0.95$ and 0.99, under the fixed candidate set scenario with three different test set sizes ($n_{0} =$ 50, 75, and 100) and the non-fixed candidate set scenario with $n_{0} = 100$, for both the targeted and untargeted methods in the soybean dataset

Scenario	Method	$n_{0}$	${\text{RErs}}\left( {n_{t}^{*} } \right) = 0.95$	${\text{RErs}}\left( {n_{t}^{*} } \right) = 0.99$
Fixed candidate set	Targeted	50	114.37 (5.07)	200.50 (5.69)
		75	116.37 (2.27)	200.67 (2.99)
		100	118.80 (2.28)	201.50 (2.53)
	Untargeted		144 (NA)	221 (NA)
Non-fixed candidate set	Targeted	100	125.87 (4.35)	216.47 (5.19)
	Untargeted	100	150.83 (1.21)	235.23 (1.22)

From Tables 2, 3 and 4, we first considered the fixed candidate set scenario. For the targeted method, the optimal training set size determined at $${\text{RErs}}\left( {n_{t}^{*} } \right)$$ = 0.95 increases approximately by two individuals on average as the test set size ($$n_{0} )$$ increases by 25 individuals, except the case that the 44 K rice dataset presented six individuals from $$n_{0} =$$ 50 to 75. The corresponding quantities at $${\text{RErs}}\left( {n_{t}^{*} } \right)$$ = 0.99 are even smaller, and the largest one is just two individuals in the 44 K rice dataset (Table 2). The optimal training set size required to predict the test set with $$n_{0} =$$ 50 was also sufficient for other larger test sets in each dataset. For a fixed $$n_{0}$$, an extra number of individuals (approximately 70, 55, and 85 individuals on average for the 44 K rice, tropical rice, and soybean datasets, respectively) are required in the training set to attain $${\text{RErs}}\left( {n_{t}^{*} } \right)$$ from 0.95 to 0.99. For the untargeted method, it generally requires a much larger training set than its targeted counterpart to achieve the same relative *r*-score. Those are approximately by 101, 20, and 28 individuals on average at $${\text{RErs}}\left( {n_{t}^{*} } \right) = 0.95$$; and 106, 11, and 21 individuals on average at $${\text{RErs}}\left( {n_{t}^{*} } \right) = 0.99$$ for the 44 K rice, tropical rice, and soybean datasets, respectively.

We then considered the non-fixed candidate set scenario. For the targeted method, an extra number of individuals (approximately 105, 80, and 91 individuals on average for the 44 K rice, tropical rice, and soybean datasets, respectively) are required in the optimal training set to attain ${\text{RErs}}\left( {n_{t}^{*} } \right)$ from 0.95 to 0.99; the corresponding quantities are 100, 67, and 85 individuals with the untargeted method. The untargeted method requires more individuals to be presented in the optimal training set, by approximately 122, 26, and 25 individuals on average for the 44 K rice, tropical rice, and soybean datasets, respectively, than the targeted method at ${\text{RErs}}\left( {n_{t}^{*} } \right)$ = 0.95; the corresponding quantities are 127, 13, and 19 individuals at ${\text{RErs}}\left( {n_{t}^{*} } \right)$ = 0.99.

**Fig. 1:**
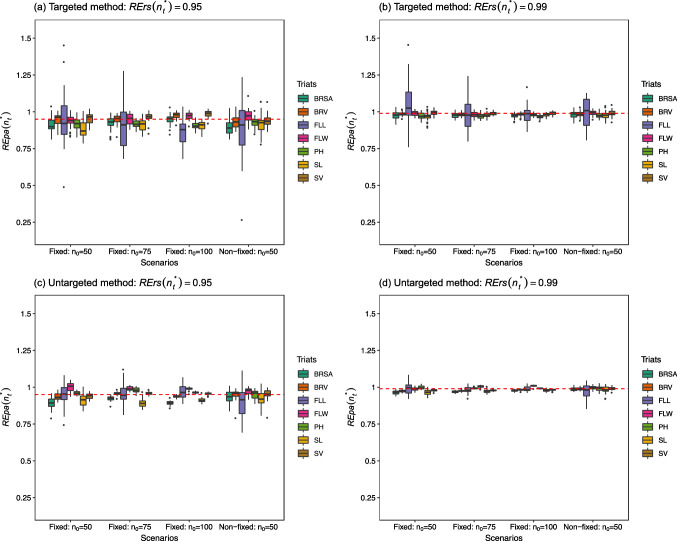
Side-by-side box plots for the ${\text{REpa}}\left( {n_{t}^{*} } \right)s$ over 30 repetitions at ${\text{RErs}}\left( {n_{t}^{*} } \right) = 0.95$ and 0.99, under a fixed candidate set scenario with three test set sizes ($n_{0} =$ 50, 75, and 100) and a non-fixed candidate set scenario with test $n_{0} = 50$ for both the targeted and untargeted methods in the 44 K rice dataset. *BRSA* Brown rice seed area, *BRV* Brown rice volume, *FLL* Flag leaf length, *FLW* Flag leaf width, *PH* Plant height, *SL* Seed length, *SV* Seed volume. The corresponding ${\text{RErs}}\left( {n_{t}^{*} } \right)$ is indicated as a red dashed line

**Fig. 2:**
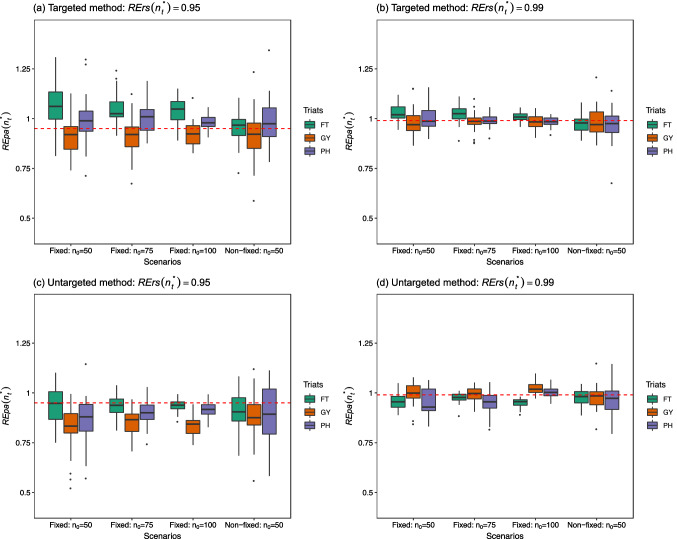
Side-by-side box plots for the ${\text{REpa}}\left( {n_{t}^{*} } \right)s$ over 30 repetitions at ${\text{RErs}}\left( {n_{t}^{*} } \right) = 0.95$ and 0.99, under a fixed candidate set scenario with three different test set sizes ($n_{0} =$ 50, 75, and 100) and a non-fixed candidate set scenario with $n_{0} = 50$, for both the targeted and untargeted methods in the tropical rice dataset. *FT* Flowering time, *GY* Grain yield, *PH* Plant height. The corresponding ${\text{RErs}}\left( {n_{t}^{*} } \right)$ is indicated as a red dashed line

**Fig. 3:**
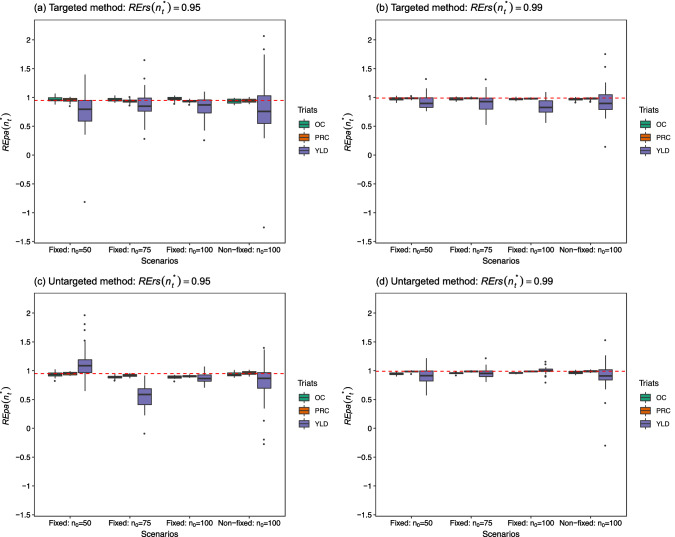
Side-by-side box plots for ${\text{REpa}}\left( {n_{t}^{*} } \right)s$ over 30 repetitions at ${\text{RErs}}\left( {n_{t}^{*} } \right) = 0.95$ and 0.99 under a fixed candidate set scenario with three different test set sizes ($n_{0} =$ 50, 75, and 100) and a non-fixed candidate set scenario with $n_{0} = 100$, for both the targeted and untargeted methods in the soybean dataset. *OC* Oil content, *PRC* Protein content, *YLD* Yield. The corresponding ${\text{RErs}}\left( {n_{t}^{*} } \right)$ is indicated as a red dashed line

The side-by-side box plots of the resulting 30 ${\text{REpa}}\left( {n_{t}^{*} } \right)$ s for the traits in each dataset are separately displayed in Figs. 1, 2 and 3. The average prediction ability over 30 $r\left( {n_{t}^{*} } \right)$ s for each trait in every dataset is displayed in Tables S1–S3 of the Supplementary Materials. We first considered the 44 K rice dataset. For a particular trait with the fixed candidate set scenario, the box plots in the four panels of Fig. 1 generally reflect the result that the larger training set size leads to more precise estimates of ${\text{REpa}}\left( {n_{t}^{*} } \right)$, i.e., a smaller dispersion of the estimates. For example, the box plot for FLL with $n_{0} =$ 100 in panel (d) appears to have a narrower spread not only than the remaining two cases of $n_{0} =$ 75 and 50 in the same panel but also all of the three cases in panels (a)–(c) with the fixed candidate set scenario. The case for a particular trait with the non-fixed candidate set scenario presented a larger dispersion than the other cases with a fixed candidate set scenario in the same panel.

Regarding the results for the tropical rice dataset, the box plots in the four panels in Fig. 2 still reflect the result that a larger training set size leads to more precise estimates of ${\text{REpa}}\left( {n_{t}^{*} } \right)$ s in a fixed trait. The median in the box plot approaches the nominal ${\text{RErs}}\left( {n_{t}^{*} } \right)$ as $n_{t}^{*}$ increases, except for the traits in panel (c) of the untargeted method at ${\text{RErs}}\left( {n_{t}^{*} } \right)$ = 0.95 compared to those in panel (a) of the targeted method at ${\text{RErs}}\left( {n_{t}^{*} } \right)$ = 0.95. Overall, these results indicate that the bias in the estimation usually improves with an increase in training set size. The case for a particular trait with the non-fixed candidate set scenario presented a relatively large dispersion than the other cases with the fixed candidate set scenario in the same panel. This result is also highlighted in Fig. 1. Finally, regarding the results for the soybean dataset in Fig. 3, the box plots in the four panels for OC and PRC show that the estimates of ${\text{REpa}}\left( {n_{t}^{*} } \right)$ s for these two traits are distributed narrowly around the nominal ${\text{RErs}}\left( {n_{t}^{*} } \right)$ values such as 0.95 or 0.99.

## Discussion

**Fig. 4:**
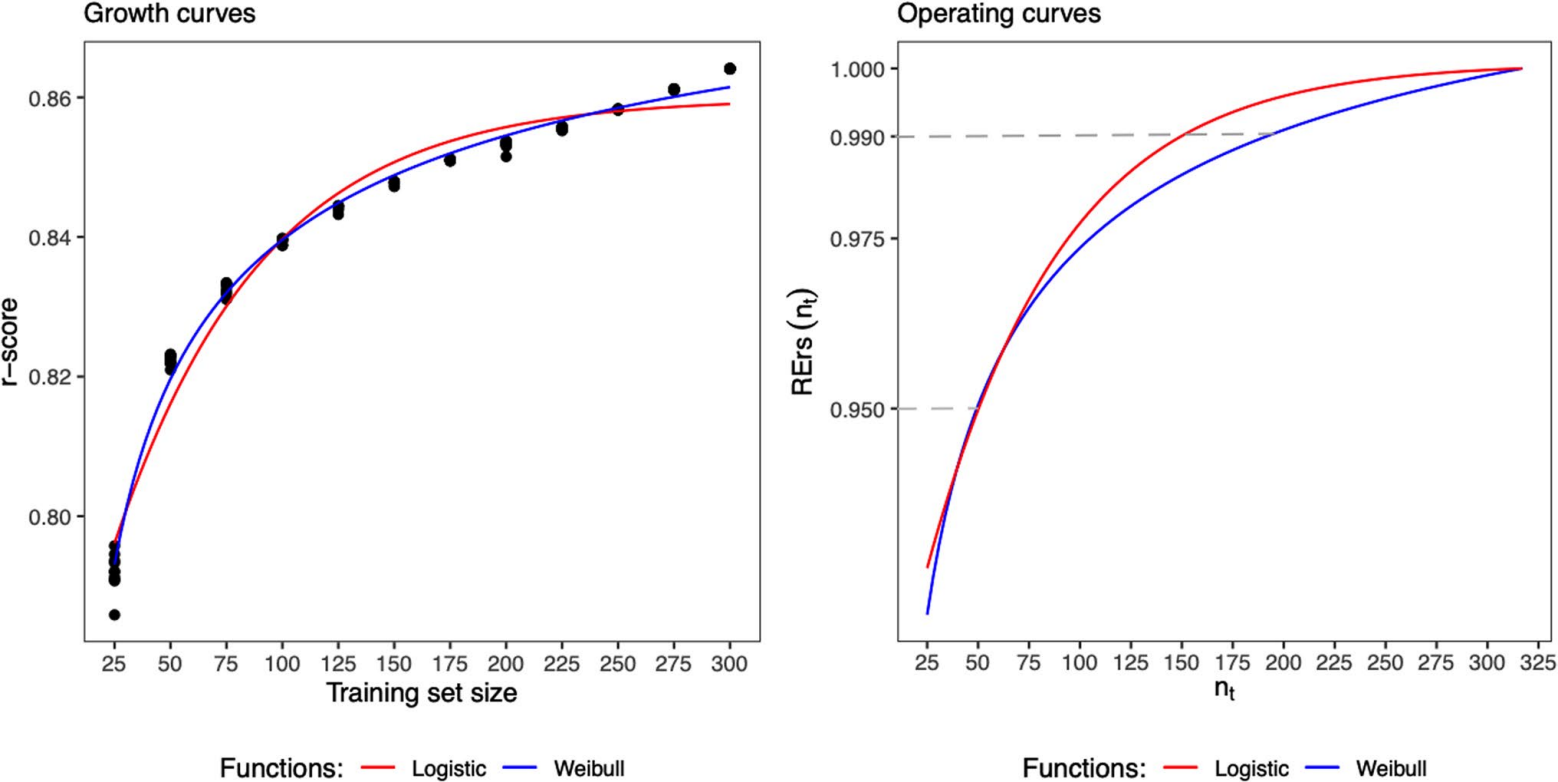
Fitted Weibull type and logistic curves (left) and operating curves (right) for the data points in the upper-left panel of Fig. S2 in the Supplementary Materials

The logistic growth curve was used in this study, mainly because it satisfies the principles of parsimony and interpretability as discussed in Ratkowsky (1993). The logistic growth curve with only three parameters provided a superior fit for almost all of the study scenarios in the three datasets, and those parameters can be used to sufficiently interpret the behavior of the observed data. However, the logistic growth function still seemed to be insufficient for some of the cases. Particularly, the targeted method under the non-fixed candidate set scenario in the 44 K rice dataset (the upper-left panel in Fig. S2 of the Supplementary Materials) had a poor fit near the maximum value of the *r*-score, which should have a significant impact on the estimation of the 95th and 99th percentile points. Therefore, we re-fitted the data points by using another four-parameter growth curve, called as Weibull type function (Ratkowsky 1983). The Weibull type function can be described as:

12 $$y = \alpha - \beta {\text{exp}}\left( { - \gamma x^{\theta } } \right)$$

where $\theta$ is an extra parameter compared to the logistic function in Eq. (7). The fitted Weibull type function together with the original logistic function, and their fitted operating curves are displayed in Fig. 4. From the figure, the Weibull type function indeed improved the fitting and resulted in a larger training set size at the 99th percentile point. In addition, the mean and standard deviation of the estimates for the parameters and the $n_{t}^{*}$ determined at ${\text{RErs}}\left( {n_{t}^{*} } \right)$ = 0.95 or 0.99 over the 30 repetitions in the same study scenario are displayed in Table 5. From which, the Weibull type function led to a training set size at ${\text{RErs}}\left( {n_{t}^{*} } \right)$ = 0.95 almost the same as the logistic function, but a much larger one at ${\text{RErs}}\left( {n_{t}^{*} } \right)$ = 0.99 (approximately by 36 genotypes on average). The above discussion implies that a more complex function could be employed when the parsimonious model does not work satisfactorily. In our experience, it becomes more challenging to set the initial values for the parameters to obtain a convergent model when performing the nonlinear least squares estimation for a more complex function.

**Table 5:** Means and standard deviations (in parentheses) of the resulting estimated parameters using logistic growth function (Eq. 7) and Weibull type function (Eq. 12), respectively, and training set sizes at ${\text{RErs}}\left( {n_{t}^{*} } \right) = 0.95$ and 0.99 over 30 repetitions under the non-fixed candidate set scenario with $n_{0} = 50$ for the targeted method in the 44 K rice dataset

Model	Parameters				Training set sizes	
	$\hat{\alpha }$	$\hat{\beta }$	$\hat{\gamma }$	$\hat{\theta }$	${\text{RErs}}\left( {n_{t}^{*} } \right) = 0.95$	${\text{RErs}}\left( {n_{t}^{*} } \right) = 0.99$
Logistic	0.8750 (0.0126)	− 2.2867 (0.1383)	0.0152 (0.0027)	–	42.03 (7.52)	147.77 (16.64)
Weibull	83.7296 (0.7111)	82.8027 (0.7022)	− 0.0045 (0.0018)	− 0.3656 (0.1428)	42.23 (6.52)	183.77 (16.38)

Spline (piecewise polynomial) regression can also be applied to perform the task of this study. However, deciding on the number and positions of the knots and the order of polynomials in each segment is not simple. In addition, the great flexibility of spline functions often makes it very easy to overfit the data when using spline regression (Montgomery and Peck 1982). Another advantage of growth curve-based regression over spline regression is that it allows a connection to be drawn between the *r*-score and the training set size in a single model across all datasets, as well as fixed and non-fixed candidate set scenarios with targeted and untargeted methods.

Although Ou and Liao (2019) found that the *r*-score was relatively robust under various values of the shrinkage parameter $\lambda$, it is not yet known how robust it is for calculating the optimal training set size $n_{t}^{*}$. We thus validated its robustness by calculating the $n_{t}^{*}$ in a fixed candidate set scenario with an untargeted method at various values of ${\uplambda }$. The results are displayed in Table 6, and they clearly show that our proposed procedure for the sample size determination can be free from the setting of the value of $\lambda$.

**Table 6:** The optimal training set size determined for the fixed candidate set scenario with the untargeted method over various values of $\lambda$ for the datasets

Dataset		$\lambda$						
		0.001	0.01	0.1	1	10	100	1000
44 K rice	0.95	138	138	138	138	138	138	138
	0.99	214	214	214	214	214	214	214
Tropical rice	0.95	131	131	130	131	131	131	131
	0.99	177	177	177	177	177	177	177
Soybean	0.95	144	144	144	144	144	144	141
	0.99	222	221	222	221	221	221	219

We considered the stratified *r*-score for the 44 K rice and soybean datasets, because their population structures could affect the training set size determination. The population structure and the clustering of the datasets are displayed in Fig. S7 of the Supplementary Materials. The results showed that the 44 K rice dataset (approximately 46% genetic variability explained by the first two PCs) had much stronger population structure than the soybean dataset (approximately 24% genetic variability explained by the first two PCs). This observation might reflect the results shown in Tables 2 and 4 that the $n_{t}^{*}$ determined at ${\text{RErs}}\left( {n_{t}^{*} } \right)$ = 0.95 or 0.99 in the 44 K rice dataset were relatively small compared with their counterpart scenarios in the soybean dataset.

Another factor that may influence the determination of the training set size is the marker density of the datasets. To explore the impact on the sample size determination, we calculated the $n_{t}^{*}$ under the fixed candidate set scenario with the untargeted method at various levels of maker density in the datasets. The subsets of markers selected from each dataset were evenly distributed over each rice or soybean chromosome. The results are displayed in Table 7, which show that the optimal training set size might decrease if the number of markers is insufficient. For example, the size was reduced from 221 to 210 genotypes in the soybean dataset, if the number of markers was reduced from $p = 2376$ to $0.25p = 594$. Chung and Liao (2020) proposed an index called the D-score for measuring genomic diversity among genotypes. We will investigate how it affects the training set size determination in a future study.

**Table 7:** Optimal training set size determined under the fixed candidate set scenario with the untargeted method over various levels of marker density in the datasets

Dataset	${\text{RErs}}\left( {n_{t}^{*} } \right)$	Marker density			
		*p*	0.75*p*	0.5*p*	0.25*p*
44 K rice	0.95	138	138	138	137
	0.99	214	214	214	214
Tropical rice	0.95	131	131	130	130
	0.99	177	177	177	177
Soybean	0.95	144	142	141	134
	0.99	221	220	218	210

Note that the numbers of SNP markers are given by $p =$ 31,401, 73,147, and 2,376 for the 44 K rice, tropical rice, and soybean datasets, respectively

As shown in Tables 2, 3 and 4, the marker-associated matrix of a dataset has a key impact on the determination of the optimal training set. In general, the use of test set information while building a training set results in much more economical phenotyping cost, in terms of the number of genotypes, than its untargeted counterpart. Moreover, larger test sets did not significantly increase the size required for the optimal training set to attain an expected accuracy (${\text{RErs}}\left( {n_{t}^{*} } \right)$ = 0.95 or 0.99). A number of additional individuals was required in the training set to attain a higher expected accuracy (${\text{RErs}}\left( {n_{t}^{*} } \right)$ is from 0.95 to 0.99), the required quantity varied with the datasets.

In this study, ${\text{REpa}}\left( {n_{t}^{*} } \right)$ of Eq. (11) was used to estimate ${\text{RErs}}\left( {n_{t}^{*} } \right)$ of Eq. (8). The factors affecting prediction ability, such as sample size, population structure, marker density, trait heritability, genetic architecture, and statistical estimation methods (Zhong et al. 2009; Zhang et al. 2019) should also influence bias and dispersion in the estimation. As shown in Figs. 1, 2 and 3, the size of the optimal training set size could be a key factor affecting both the bias and dispersion in the estimation, which can be improved as the size $n_{t}^{*}$ increases. In particular, the box plots for FLL in Fig. 1 of the 44 K rice dataset and those for YLD in Fig. 3 of the soybean dataset were found to be relatively dispersed compared with the remaining traits in the same dataset. From Tables S1 and S3, these two traits have the lowest prediction ability (FLL: 0.35–0.51; YLD: 0.16–0.28) among the traits in the same dataset. This may be partially due to their relative low trait heritability (FLL: 0.0354; YLD: 0.0355). The trait heritability estimated from all available phenotypic values for each trait-dataset combination based on the GBLUP model of Eq. (4) is displayed in Table S4 of the Supplementary Materials.

In addition, a targeted optimization usually outperformed its untargeted counterpart, as expected. Most of the resulting ${\text{REpa}}\left( {n_{t}^{*} } \right)$ over the 30 repetitions for all the data-trait combinations were smaller than 1, meaning that $r\left( {n_{t}^{*} } \right) < r\left( {n_{c} } \right)$. However, there were still some cases of ${\text{REpa}}\left( {n_{t}^{*} } \right) > 1$, with the result that $r\left( {n_{t}^{*} } \right) > r\left( {n_{c} } \right)$ for these cases. This interesting result indicated that the optimal training set, which excludes irrelevant candidates, can enhance the prediction ability and reduce the size of training set. An optimal training set with a sufficient size might provide more powerful prediction ability than the entire candidate set for particular dataset-trait combinations.

Based on the GBLUP model of Eq. (4), the CD criterion can be treated as an index for measuring the correlation between the GEBVs and the true genotypic values (Laloë 1993; Rincent et al. 2012). This is similar to the concept of the developing *r*-score, which is based on the correlation between the GEBVs and the phenotypic values. Therefore, the CD criterion can be a promising alternative for incorporation into our proposed procedure. We are currently investigating this interesting issue and will present the results in a future communication. Another useful optimization criterion of PEV, proposed by Akdemir et al. (2015), is anticipated to produce a decreasing function with the size of the training set, so suitable declining curves (Kawabata and DeFrank 1994) might be applied to the sample size determination.

An R function for executing the proposed approach, called SSDFGP, is available from the package TSDFGS (Ou 2022). A user can install the package from the R office repository CRAN or GitHub. Our proposed approach should prove useful to determine the composition and size of an optimal training set for genomic selection.

## Supplementary Information

Below is the link to the electronic supplementary material.

Supplementary file1 (DOCX 715 KB)

## Data Availability

All the datasets used in this article are freely accessible and can be downloaded from the cited references.
