# Supplementary material for: Sample size determination for training set optimization in genomic prediction
Source: Theor Appl Genet. 2023 Mar 13;136(3):57. doi: 10.1007/s00122-023-04254-9 (PMC10011335; doi:10.1007/s00122-023-04254-9)
Supplement: Supplementary file 1 — Supplementary file1 (DOCX 715 KB) [file 122_2023_4254_MOESM1_ESM.docx]

**Supplementary Materials**

Figure S1. Fitted logistic growth curves (left) and operating curves (right) for both (1) the targeted method with three different test sizes ($n_{0}=$ 50, 75 and 100), and (2) the untargeted method under the fixed candidate set scenario in the 44K rice dataset.

Figure S2. Fitted logistic growth curves (left) and operating curves (right) for both (1) the targeted method with $n_{0}=$50, and (2) the untargeted method under the non-fixed candidate set scenario in the 44K rice dataset.

Figure S3. Fitted logistic growth curves (left) and operating curves (right) for both (1) the targeted method with three different test sizes ($n_{0}=$ 50, 75 and 100), and (2) the untargeted method under the fixed candidate set scenario in the tropical rice dataset.

Figure S4. Fitted logistic growth curves (left) and operating curves (right) for both (1) the targeted method with $n_{0}=$50, and (2) the untargeted method under the non-fixed candidate set scenario in the tropical rice dataset.

Figure S5. Fitted logistic growth curves (left) and operating curves (right) for both (1) the targeted method with three different test sizes ($n_{0}=$ 50, 75 and 100), and (2) the untargeted method under the fixed candidate set scenario in the soybean dataset.

Figure S6. Fitted logistic growth curves (left) and operating curves (right) for both (1) the targeted method with $n_{0}=$100, and (2) the untargeted method under the non-fixed candidate set scenario in the soybean dataset.

Figure S7. (a) Plot of the top four principle components with 31,401 SNP markers for 367 accessions in 44K rice dataset. The five subpopulations are admixed group (ADMIX), aromatic (AROMATIC), *aus* (AUS), *indica* (IND), *temperate japonica* (TEJ), and *tropical japonica* (TRJ). (b) Plot of the first two principle components with 73,147 SNP markers for 328 accessions in tropical rice dataset. (c) Plot of the first two principle components with 2,376 SNP markers for 401 accessions in soybean dataset.

Table S1. Means and standard deviations (in parentheses) of prediction abilities of the seven traits over 30 repetitions at $RErs\left( n_{t}^{*} \right)=0.95$ and 0.99, under the fixed candidate set scenario with three different test set sizes ($n_{0}=$50, 75 and 100) and the non-fixed candidate set scenario with $n_{0}=50$, for both the targeted and untargeted methods in the 44K rice dataset.

|  |  |  |  |  |  | Trait^*^ |  |  |  |
| --- | --- | --- | --- | --- | --- | --- | --- | --- | --- |
| Method | $RErs\left( n_{t}^{*} \right)$ | Scenario | BRSA | BRV | FLL | FLW | PH | SL | SV |
| Targeted | $0.95$ | Fixed: $n_{0}$=50 | 0.67 (0.07) | 0.76 (0.06) | 0.39 (0.08) | 0.69 (0.06) | 0.72 (0.05) | 0.64 (0.05) | 0.75 (0.06) |
|  |  | Fixed: $n_{0}$=75 | 0.69 (0.04) | 0.77 (0.04) | 0.36 (0.06) | 0.71 (0.05) | 0.73 (0.04) | 0.67 (0.04) | 0.76 (0.04) |
|  |  | Fixed: $n_{0}$=100 | 0.70 (0.03) | 0.78 (0.02) | 0.35 (0.05) | 0.72 (0.02) | 0.72 (0.03) | 0.67 (0.02) | 0.78 (0.02) |
|  |  | Non-fixed: $n_{0}$=50 | 0.71 (0.07) | 0.78 (0.05) | 0.47 (0.11) | 0.73 (0.06) | 0.75 (0.05) | 0.71 (0.06) | 0.78 (0.05) |
|  | 0.99 | Fixed: $n_{0}$=50 | 0.72 (0.06) | 0.79 (0.05) | 0.43 (0.08) | 0.74 (0.04) | 0.77 (0.05) | 0.71 (0.05) | 0.78 (0.05) |
|  |  | Fixed: $n_{0}$=75 | 0.73 (0.03) | 0.79 (0.02) | 0.39 (0.05) | 0.73 (0.04) | 0.77 (0.03) | 0.72 (0.03) | 0.78 (0.03) |
|  |  | Fixed: $n_{0}$=100 | 0.72 (0.03) | 0.79 (0.02) | 0.40 (0.03) | 0.73 (0.01) | 0.77 (0.02) | 0.73 (0.02) | 0.78 (0.02) |
|  |  | Non−fixed: $n_{0}$=50 | 0.77 (0.06) | 0.83 (0.05) | 0.53 (0.09) | 0.75 (0.06) | 0.79 (0.04) | 0.75 (0.05) | 0.82 (0.05) |
| Untargeted | 0.95 | Fixed: $n_{0}$=50 | 0.66 (0.07) | 0.75 (0.05) | 0.40 (0.07) | 0.74 (0.04) | 0.76 (0.04) | 0.68 (0.07) | 0.74 (0.05) |
|  |  | Fixed: $n_{0}$=75 | 0.69 (0.03) | 0.77 (0.03) | 0.39 (0.07) | 0.74 (0.03) | 0.78 (0.03) | 0.66 (0.04) | 0.76 (0.03) |
|  |  | Fixed: $n_{0}$=100 | 0.66 (0.03) | 0.75 (0.02) | 0.39 (0.04) | 0.73 (0.02) | 0.77 (0.02) | 0.68 (0.02) | 0.75 (0.02) |
|  |  | Non-fixed: $n_{0}$=50 | 0.74 (0.06) | 0.80 (0.06) | 0.48 (0.09) | 0.73 (0.06) | 0.77 (0.05) | 0.70 (0.06) | 0.79 (0.06) |
|  | 0.99 | Fixed: $n_{0}$=50 | 0.71 (0.06) | 0.78 (0.05) | 0.41 (0.08) | 0.74 (0.04) | 0.79 (0.04) | 0.71 (0.06) | 0.77 (0.05) |
|  |  | Fixed: $n_{0}$=75 | 0.73 (0.03) | 0.79 (0.03) | 0.40 (0.06) | 0.74 (0.03) | 0.80 (0.03) | 0.72 (0.03) | 0.77 (0.03) |
|  |  | Fixed: $n_{0}$=100 | 0.73 (0.02) | 0.79 (0.02) | 0.40 (0.03) | 0.75 (0.01) | 0.79 (0.02) | 0.73 (0.02) | 0.77 (0.02) |
|  |  | Non-fixed: $n_{0}$=50 | 0.78 (0.05) | 0.83 (0.05) | 0.51 (0.08) | 0.75 (0.06) | 0.80 (0.04) | 0.75 (0.05) | 0.82 (0.05) |

* BRSA: brown rice seed area; BRV: brown rice volume; FLL: flag leaf length; FLW: flag leaf width; PH: plant height; SL: seed length; SV: seed volume

Table S2. Means and standard deviations (in parentheses) of prediction abilities of the three traits over the 30 repetitions at $RErs\left( n_{t}^{*} \right)=0.95$ and 0.99, under the fixed candidate set scenario with three different test set sizes ($n_{0}=$50, 75 and 100) and the non-fixed candidate set scenario with $n_{0}=50$, for both the targeted and untargeted methods in the tropical rice dataset.

|  |  |  |  | Trait^*^ |  |
| --- | --- | --- | --- | --- | --- |
| Method | $RErs\left( n_{t}^{*} \right)$ | Scenario | FT | GY | PH |
| Targeted | $0.95$ | Fixed: $n_{0}$=50 | 0.50 (0.07) | 0.42 (0.11) | 0.45 (0.08) |
|  |  | Fixed: $n_{0}$=75 | 0.51 (0.06) | 0.39 (0.06) | 0.45 (0.05) |
|  |  | Fixed: $n_{0}$=100 | 0.51 (0.04) | 0.40 (0.05) | 0.46 (0.04) |
|  |  | Non-fixed: $n_{0}$=50 | 0.53 (0.08) | 0.46 (0.09) | 0.44 (0.10) |
|  | 0.99 | Fixed: $n_{0}$=50 | 0.48 (0.08) | 0.44 (0.11) | 0.46 (0.08) |
|  |  | Fixed: $n_{0}$=75 | 0.49 (0.06) | 0.42 (0.07) | 0.45 (0.06) |
|  |  | Fixed: $n_{0}$=100 | 0.50 (0.04) | 0.43 (0.05) | 0.46 (0.04) |
|  |  | Non−fixed: $n_{0}$=50 | 0.54 (0.08) | 0.51 (0.09) | 0.43 (0.11) |
| Untargeted | 0.95 | Fixed: $n_{0}$=50 | 0.44 (0.09) | 0.38 (0.12) | 0.40 (0.09) |
|  |  | Fixed: $n_{0}$=75 | 0.45 (0.07) | 0.37 (0.07) | 0.41 (0.06) |
|  |  | Fixed: $n_{0}$=100 | 0.46 (0.04) | 0.37 (0.05) | 0.43 (0.04) |
|  |  | Non-fixed: $n_{0}$=50 | 0.50 (0.09) | 0.46 (0.10) | 0.40 (0.12) |
|  | 0.99 | Fixed: $n_{0}$=50 | 0.45 (0.08) | 0.45 (0.12) | 0.44 (0.08) |
|  |  | Fixed: $n_{0}$=75 | 0.47 (0.07) | 0.43 (0.07) | 0.43 (0.07) |
|  |  | Fixed: $n_{0}$=100 | 0.47 (0.04) | 0.45 (0.05) | 0.47 (0.04) |
|  |  | Non-fixed: $n_{0}$=50 | 0.54 (0.08) | 0.50 (0.10) | 0.43 (0.11) |

*FT: flowering time; GY: grain yield; PH: plant height

Table S3. Means and standard deviations (in parentheses) of prediction abilities of the three traits over the 30 repetitions at $RErs\left( n_{t}^{*} \right)=0.95$ and 0.99, under the fixed candidate set scenario with three different test set sizes ($n_{0}=$50, 75 and 100) and the non-fixed candidate set scenario with $n_{0}=100$, for both the targeted and untargeted methods in the soybean dataset.

|  |  |  |  | Trait^*^ |  |
| --- | --- | --- | --- | --- | --- |
| Method | $RErs\left( n_{t}^{*} \right)$ | Scenario | OC | PRC | YLD |
| Targeted | $0.95$ | Fixed: $n_{0}$=50 | 0.66 (0.06) | 0.76 (0.05) | 0.24 (0.13) |
|  |  | Fixed: $n_{0}$=75 | 0.68 (0.04) | 0.74 (0.04) | 0.24 (0.09) |
|  |  | Fixed: $n_{0}$=100 | 0.69 (0.04) | 0.74 (0.02) | 0.23 (0.06) |
|  |  | Non-fixed: $n_{0}$=100 | 0.66 (0.05) | 0.75 (0.04) | 0.16 (0.09) |
|  | 0.99 | Fixed: $n_{0}$=50 | 0.67 (0.07) | 0.79 (0.04) | 0.28 (0.11) |
|  |  | Fixed: $n_{0}$=75 | 0.68 (0.03) | 0.78 (0.03) | 0.26 (0.08) |
|  |  | Fixed: $n_{0}$=100 | 0.68 (0.03) | 0.78 (0.02) | 0.23 (0.05) |
|  |  | Non-fixed: $n_{0}$=100 | 0.68 (0.04) | 0.78 (0.04) | 0.19 (0.09) |
| Untargeted | 0.95 | Fixed: $n_{0}$=50 | 0.64 (0.07) | 0.76 (0.05) | 0.33 (0.11) |
|  |  | Fixed: $n_{0}$=75 | 0.62 (0.04) | 0.72 (0.04) | 0.16 (0.08) |
|  |  | Fixed: $n_{0}$=100 | 0.63 (0.04) | 0.72 (0.02) | 0.24 (0.04) |
|  |  | Non-fixed: $n_{0}$=100 | 0.66 (0.04) | 0.76 (0.05) | 0.17 (0.09) |
|  | 0.99 | Fixed: $n_{0}$=50 | 0.65 (0.06) | 0.79 (0.05) | 0.28 (0.11) |
|  |  | Fixed: $n_{0}$=75 | 0.67 (0.03) | 0.78 (0.03) | 0.27 (0.07) |
|  |  | Fixed: $n_{0}$=100 | 0.68 (0.03) | 0.78 (0.02) | 0.27 (0.04) |
|  |  | Non-fixed: $n_{0}$=100 | 0.68 (0.05) | 0.79 (0.03) | 0.19 (0.09) |

*OC: oil content; PRC: protein content; YLD: yield

Table S4. The estimates of variance components and heritability ($h^{2}$) for each trait-dataset combination based on GBLUP model. The$h^{2}$ is calculated by $\frac{\hat{\sigma}_{g}^{2}}{\hat{\sigma}_{g}^{2}+\hat{\sigma}_{\varepsilon}^{2}}$.

|  | 44K rice dataset | | | | | | | Tropical rice dataset | | | Soybean dataset | | |
| --- | --- | --- | --- | --- | --- | --- | --- | --- | --- | --- | --- | --- | --- |
|  | Trait^1^ | | | | | | | Trait^2^ | | | Trait^3^ | | |
|  | BRSA | BRV | FLL | FLW | PH | SL | SV | FT | GY | PH | OC | PRC | YLD |
| $\hat{\sigma}_{g}^{2}$ | 0.0004 | 0.0218 | 0.4603 | 0.0005 | 4.2386 | 0.0193 | 0.0008 | 0.0498 | 907.6542 | 0.1793 | 0.0586 | 0.1818 | 21.9362 |
| $\hat{\sigma}_{\varepsilon}^{2}$ | 0.0003 | 0.1413 | 12.5130 | 0.0171 | 78.4091 | 0.0125 | 0.0039 | 4.7183 | 77974.1159 | 13.6010 | 0.0494 | 0.1302 | 596.6084 |
| $H^{2}$ | 0.5470 | 0.1334 | 0.0354 | 0.0281 | 0.0513 | 0.6075 | 0.1688 | 0.0104 | 0.0115 | 0.0130 | 0.5426 | 0.5826 | 0.0355 |

^1^ BRSA: brown rice seed area; BRV: brown rice volume; FLL: flag leaf length; FLW: flag leaf width; PH: plant height; SL: seed length; SV: seed volume

^2^ FT: flowering time; GY: grain yield; PH: plant height

^3^ OC: oil content; PRC: protein content; YLD: yield
